# The role of primary school composition in affective decision-making: a prospective cohort study

**DOI:** 10.1007/s00127-022-02252-8

**Published:** 2022-05-10

**Authors:** E. Papachristou, E. Flouri, H. Joshi

**Affiliations:** UCL Institute of Education, University College London, 25 Woburn Square, London, WC1H 0AA UK

**Keywords:** Childhood, Decision-making, Millennium Cohort Study, School composition

## Abstract

**Purpose:**

School-level characteristics are known to be associated with pupils’ academic and cognitive ability but also their socioemotional development. This study examines, for the first time, whether primary school characteristics are associated with pupils’ affective decision-making too.

**Methods:**

The sample included 3,141 children participating in the Millennium Cohort Study with available data on their school’s characteristics, according to the National Pupil Database, at age 7 years. Decision-making was measured using the Cambridge Gambling Task at age 11 years. We modelled data using a series of sex-stratified linear regression analyses of decision-making (risk‐taking, quality of decision‐making, risk adjustment, deliberation time, and delay aversion) against four indicators of school composition (academic performance and proportions among pupils who are native speakers of English, are eligible for free school meals and have special educational needs).

**Results:**

After adjustment for individual and family-level confounding, schools with a higher average academic performance showed more delay aversion among males, and among females, higher deliberation time and lower risk-taking. Schools with proportionally more native English speakers had higher deliberation time among males. Schools with proportionally more pupils eligible for free school meals showed lower scores on quality of decision-making among males. Schools with proportionally more children with special educational needs showed better quality of decision-making among males and lower risk-taking among females.

**Conclusion:**

The findings of this study can be used to target support for primary schools. Interventions aiming to support lower-achieving schools and those with less affluent intakes could help to improve boys’ affective decision-making.

School compositional and functional characteristics such as teaching quality, school size, and average socioeconomic status (SES) of the student body are known to be associated with individual pupils’ academic performance [1, 2] and behavioural and emotional outcomes [3, 4]. This study of primary school children aims to examine for the first time whether school characteristics are associated with individual pupils’ affective decision-making too. Affective decision-making (henceforth decision-making) is best defined as the strategic process of choice under risk and the result of the interaction between ‘rational’ and ‘emotional’ processes [5]. Most commonly measured with gambling tasks, decision-making is, if poor, a correlate of problem behaviours [6, 7] and low cognitive ability [8, 9], but also a risk marker for future risk-taking behaviours [10] and psychopathology [11, 12] in children and adolescents. If school characteristics were found to be significantly associated with individual pupils’ decision-making as early as at primary school, the findings of this study could have significant implications for educational policy by revealing what type of school context may have a role in shaping adaptive reward responses. Given that decision-making is significantly associated with socioemotional development and academic ability in childhood, as well as a wide range of outcomes in adolescence, such efforts would also have the potential to aid academic performance and to alleviate some of the burden associated with mental ill-health in children [13] and beyond. Moreover, this study could help to identify those school characteristics that exacerbate educational inequalities. Various school-level risk concerns, such as poor school climate and low social capital, have been shown to be more common among pupils from more deprived social backgrounds [14, 15]. Since these school characteristics are associated, in turn, with individual pupils’ outcomes across many domains, this study’s findings could provide evidence on the ‘double disadvantage’ that pupils from disadvantaged (and certain BAME) backgrounds are facing by attending schools of relative poorer quality in key aspects.

Ethnic diversity, a school’s average academic performance, and the average socioeconomic status of the parents of its pupils are the most examined school-level characteristics in relation to individual pupils’ cognitive and socioemotional outcomes. Ethnically diverse schools are thought to be cognitively stimulating contexts by enforcing interactions with peers from other ethnicities [16–18]. According to this, such interactions require pupils to encounter experiences and demands that they cannot completely understand or easily meet, thus promoting cognitive growth and improved academic performance. School-level academic performance is negatively associated with both delinquent behaviour [19, 20] and emotional symptoms [21]. Although the reasons are not fully understood, a plausible explanation is that academically high-performing schools encourage spending more time on doing homework. Time spent constructively on homework is known to be negatively related to cigarette smoking, illicit drug use, and delinquency [22]. Finally, regarding the average socioeconomic status of the pupils’ parents at the school-level, there is much evidence for its role in individual pupils’ academic outcomes, and some recent evidence suggesting links with their socioemotional outcomes as well. For example, a recent UK study showed a significant positive association between the school’s proportion of pupils who are eligible for free school meals (FSM) and externalising problem trajectories in childhood and adolescence, over and above the effect of FSM eligibility of the individual child [4]. Yet, there are also null results with respect to associations between school-level poverty and individual-level misbehaviour [23]. Stewart’s (2003) findings, for example, suggest that economic inequality within the school, rather than school-level SES, might be a better predictor of misbehaviour [23].

Although these school compositional characteristics have not been linked to pupils’ decision-making, their individual-level equivalents have. Poor socioeconomic circumstances in childhood are shown to have a long-standing impact on a preference for immediate, smaller rewards over larger rewards delayed in time, in adulthood [24]. Among adolescents, those from ethnic minorities, and those with low SES in particular, are more likely to show diminished impulse control relative to the majority group [25]. Finally, poor academic performance is also associated with poorer decision-making ability and increased risk-taking in adolescence [26], potentially because higher intellectual ability, a strong correlate of academic performance, can act as a protective factor for risk-taking behaviours by aiding better understanding of the potential consequences of risk-taking.

The aim of the study was to fill the gap about the role of school composition in individual decision-making by exploring to what extent SES, ethnicity and academic performance measured at the school-level may be associated with decision-making in primary school children. The identification of school-level risk factors for poor decision-making in childhood can inform legislation on admission policy for primary schools and identify targets for intervention, but also help parents with school choice, a much debated topic in England [27]. A potential reason for this apparent gap in the literature has been a lack of administrative data capturing both individual and school-level variables. A unique opportunity has arisen to test such hypotheses using data of the Millennium Cohort Study (MCS) [28, 29], described in more detail below, which have been linked to the National Pupil Database (NPD). The NPD is a rich dataset of the Department for Education (DfE) in England collected directly from schools, local authorities, and awarding bodies, alongside data from annual school censuses.

In line with findings of studies on the role of school composition for children’s broad emotional and behavioural outcomes, we hypothesised for this study that children in primary schools with lower proportions of pupils on FSM (as a proxy of SES), in schools that are more ethnically diverse and in schools with higher average academic performance would show better decision-making. Specifically, we hypothesised that children in such schools would demonstrate better quality and less impulsive decision-making, as measured by the Cambridge Gambling Task (CGT), administered in MCS at age 11. Due to a scarcity of evidence, we additionally investigated in an exploratory manner the role of the school’s percentage of native English speakers and the percentage of children with special educational needs (SEN) in decision-making. The CGT is a validated gambling task measuring different aspects of decision-making, for example risky/rational choices, betting behaviour, risk adjustment and deliberation time, outside a learning context [30–32].

## Methods

### Sample

We used data from the Millennium Cohort Study (MCS), a population-based cohort of children born in the UK over 12 months from 1 September 2000 [29]. The children in the MCS were around 9 months old at Sweep 1, and 3, 5, 7, 11 and 14 years old at Sweeps 2–6, respectively. At the six sweeps, the numbers of productive families were 18,522, 15,590, 15,246, 13,857, 13,287 and 11,714. Ethical approval was gained from NHS Multi-Centre Ethics Committees. Parents gave informed consent before interviews took place, and from age 11 children gave assent.

**Fig. 1:**
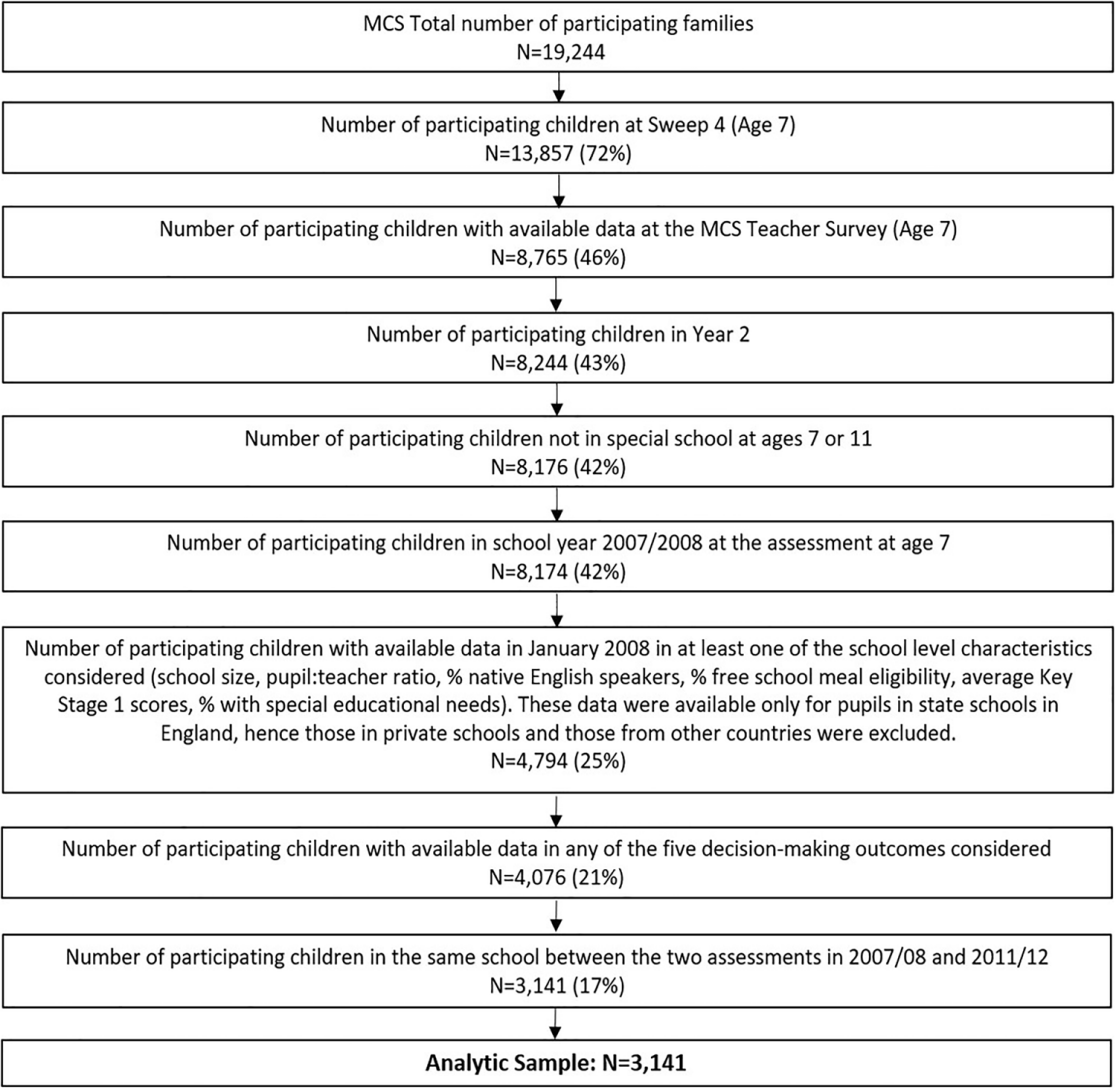
Flow chart of the study

When the children were aged 7 years, information was also collected from the cohort children’s class teachers using a self-completion postal questionnaire [33]. In total, 7,235 teachers in 4,969 schools were contacted to take part in the survey. Of those, 5,364 teachers (74.1%) from 3,981 schools (80.1%) completed and returned a questionnaire for 8,876 children. Ethical approval for the teacher survey was given by the Northern and Yorkshire Multi-Centre Research Ethics Committee (MREC) of the NHS [34]. Further approvals were sought and given for carrying out the survey in individual UK countries. For England, the teacher survey was approved by the Star Chamber in the Department for Children, Schools and Families; for Wales, by the Schools Workforce Advisory Panel; for Scotland, by the Directors of Education in the Local Educational Authorities; and for Northern Ireland, no formal approval was needed. We used data from MCS sweeps 4 (around age 7) and 5 (around age 11) [35, 36]. The flow chart illustrates the process followed to derive the analytic sample of this study (*n* = 3,141) and the associated attrition rate (Fig. 1). We count only one child per family (i.e. singletons and first-born twins or triplets). Our analytic sample included those with available information on at least one of the school compositional and structural characteristics (available in MCS only for England) considered at age 7 and with available data on at least one of the decision-making outcomes on the CGT. School-level information was obtained from the School Data Unit at the Department for Education and linked to data available from the MCS. Since that information was about pupils in state schools in England, those from the other three UK countries, as well as those in private schools, had to be excluded. We measured academic performance with Key Stage 1 (KS1) assessment data, collected at the end of Year 2 (normally the year in which pupils reach age 7), and, therefore, we also excluded two children who were not in Year 2 during that academic year. We also excluded children who changed schools between the assessments to set apart potential changes in the school compositional and functional characteristics considered in this study. Finally, we excluded children attending special schools at ages 7 or 11.

## Measures

### Cambridge gambling task

*Decision-making* at age 11 years was assessed with the CGT, which measures decision-making under risk. The CGT is a neurocognitive measure proven sensitive to deficits in reward-based decision-making, and is considered a relatively pure measure of reward-based decision-making with explicit outcome probabilities. Disadvantageous CGT outcomes have been associated with mental health problems in childhood and adolescence beyond the obvious cases of gambling and substance abuse, for example depression [37, 38], autism [39], and suicide risk [40] suggesting adequate criterion validity in youth. The task was administered using built-in touchscreens on interviewers’ computer assisted personal interview (CAPI) machines in the homes of the MCS cohort members as part of the main interview. In a series of five stages the MCS participant is presented with a row of 10 red or blue boxes across the top of the screen, appearing in varying combinations. During the first stage (decision-only) the participant is asked to guess whether a yellow token is hidden in a red or a blue box. In the remaining four (gambling) stages the participant must additionally select a portion of 100 points given to them at the beginning of the trial to gamble on their confidence in the location of the token. The ratios of red:blue boxes vary from 1:9 to 9:1 in pseudorandom order. Thus, the odds of guessing correctly are presented explicitly by varying the ratios of colours among boxes that may contain the hidden token. Participants are informed that correct bets will be added onto their points score (and incorrect ones will be taken away) and that they should try to win as many points as possible. They are asked to bet some proportion of their points (between 5 and 95%) on the certainty of their decision by selecting from an array of possible bets presented in ascending and descending sequences. Two of the gambling stages are practice sessions so that participants’ performance is ultimately assessed by the last two gambling stages. The CGT produces six outcome measures. *Delay aversion* is the time participants are prepared to wait to place a higher or lower bet. *Deliberation time* is the mean time (in milliseconds) taken to make a response on box colour. It is the latency from the presentation of the coloured boxes to bet choice. *Quality of decision-making* is the mean proportion of trials where the participant selects the most probable colour. It is the tendency to bet on the more likely outcome. *Risk adjustment* is the extent to which betting behaviour is moderated by the ratio of boxes, and reflects the tendency to stake higher bets on favourable compared to unfavourable trials. *Risk taking* is the mean proportion of points bet on trials where the most probable colour was chosen. Higher scores reflect higher reward sensitivity (or lower sensitivity to punishment). *Overall proportion bet* is the mean proportion of points bet across all trials.

### School compositional and structural characteristics

School compositional characteristics applied to state-maintained schools during January 2008 (corresponding with MCS sweep 4, age 7) and were banded into deciles based on all primary schools in England. School-average KS1 scores were averaged for English, Maths and Science across pupils. School socioeconomic composition was based on the percentage of pupils who were eligible for FSM. Pupils are entitled to FSM if their parents receive certain means-tested benefits or tax credits, subject to a gross household income ceiling. Additional school characteristics included the percentage of pupils whose first language was known or believed to be English and the percentage of children with special educational needs (SEN; SEN statistics exclude a minority whose needs are already being addressed through a formal ‘statement’ of their needs). We also considered two structural school characteristics, the headcount of pupils—as a measure of school size—and the pupil:teacher ratio which was computed by dividing the pupil headcount by the number of teachers. All sensitive data were linked with MCS data in a secure environment using the unique anonymised reference number of each child’s school.

### Covariates

To minimise confounding we adjusted for individual and family-level covariates at child’s age 7 or 11 years. Information on individual *academic performance* (measured with the child’s KS1 average score) and *FSM eligibility* at child’s age 7 years was collected during the January 2008 census and obtained from the NPD. Other covariates assessed at child’s age 7 years included *SEN status* (yes or no)*, ethnicity* (White or Non-White), *maternal education* (university degree or not), *family structure* (living with both biological parents or not), *urbanicity* (residing in an urban area or not), and *maternal psychological distress* [using the Kessler K6 [41], a validated six-question instrument estimating the prevalence of serious mental illness in general population samples [42]]. We also adjusted for internalising and externalising problems using the parent-reported Strengths and Difficulties Questionnaire (SDQ) at age 7, a short, psychometrically valid and widely used behavioural screening tool [43]. Finally, we controlled for pubertal status at the time of measurement of the CGT (around age 11 years) with the parent’s report of whether or not there was breast growth or menstruation or hair on body (for females), and voice change or facial hair or hair on body (for males).

### Statistical analysis

Analyses were performed in Stata/SE 14.2 [44] and Mplus 7.4 [45]. First, we examined the baseline sample and school characteristics in the analytic sample. Next, we calculated Spearman’s correlation coefficients for the bivariate associations between the primary school characteristics. Finally, in view of the large gender differences in decision-making measured using gambling tasks [46, 47], we ran two sets of sex-stratified multiple regression analyses. (By sex-stratifying the analyses we eliminated sex as a confounder for the relationship between school-level characteristics and decision-making.) In the first (Model A), we estimated the prospective associations between the school-level characteristics (age 7) and the five decision-making outcomes (age 11). We excluded *overall proportion bet* from all analyses and considered only the remaining five decision-making outcomes in light of its very high correlation with risk-taking in the analytic sample (*r *= 0.97, *p* < 0.001). In the second (Model B), we further adjusted the models for the individual and family-level covariates. Multicollinearity between the covariates was tested using variance inflation factor (VIF) values [VIF values > 4 indicate multicollinearity]. Missing data on covariates (and on outcomes where applicable; see Table 1) were handled using full information maximum likelihood which has been shown to produce more unbiased parameter estimates and standard errors compared to other multiple imputation techniques. All outcomes were considered in the same regression models (Mplus can accommodate multiple dependent variables in a single analytic step), thus keeping the associated Type I error rate low. Despite the apparent nested structure of the data (pupils clustered within schools) the degree of clustering by school in MCS was too low to run multilevel regression models instead of general multiple linear regression models. The cohort members in the analytic sample attended a total of 1,490 schools, each providing information for 2.1 cohort children on average. Of all schools, 67% were attended by one cohort member only, 10% by two, and only the remaining 27% by three or more cohort members. We also calculated the design effect associated with the degree of clustering of pupils within school in the analytic sample. The design effect is a measure of effect size for multilevel models and is a function of the intraclass correlation (ICC) and the average cluster size. It is calculated as ‘1 + (average cluster size—1)*ICC’. Design effects greater than 2 indicate that the clustering in the data needs to be taken into account during estimation [48]. The ICC for males and females for the 5 decision-making outcomes considered in our study ranged from 1.79e–12% to 8.9%. The resulting design effect ranged from 1 to 1.1 suggesting that the clustering in the data does not need to be taken into account during estimation. Analyses were run using the maximum likelihood with robust standard errors (MLR) estimator which can account for the skewed distribution of variables. We also applied attrition and stratification weights and cluster points to account for the disproportionately stratified and clustered design of MCS [49].

**Table 1:** Baseline sample characteristics (age 7 in around 2008, unless otherwise specified), school compositional and structural characteristics (in January 2008), and decision-making outcomes (age 11) of the analytic sample (see flow chart (Fig. 1)) (*N* = 3,141) (unweighted data)

Continuous variables	Males (*N *= 1,545; 49%)	Females (*N* = 1,596; 51%)	*p* value ^a^	Analytic sample (3,141; 100%)	
	Mean (Standard Deviation)	Mean (Standard Deviation)		Mean (Standard Deviation)	% Missing data
Delay aversion	.312 (.215)	.259 (.247)	<0 .001	.285 (.234)	0.3
Deliberation time	3,197.271 (1,169.437)	3,403.038 (1,428.553)	< 0.001	3,301.825 (1,311.367)	0.0
Quality of decision-making	.824 (.158)	.830 (.168)	0.26	.827 (.163)	0.0
Risk adjustment	.756 (1.021)	.745 (1.023)	0.76	.750 (1.022)	0.0
Risk taking	.573 (.155)	.488 (.161)	<0 .001	.530 (.164)	0.0
Internalising problems at 7	2.716 (2.856)	2.673 (2.628)	0.67	2.695 (2.742)	2.1
Externalising problems at 7	4.999 (3.502)	3.953 (3.101)	<0 .001	4.468 (3.344)	2.1
Average Key Stage 1 score	5.531 (2.993)	5.861 (2.760)	0.002	5.699 (2.881)	9.3
Maternal psychological distress	2.918 (3.650)	3.016 (3.662)	0.46	2.968 (3.656)	4.3
School size (deciles)	6.952 (2.620)	7.09 (2.573)	0.15	7.020 (2.597)	1.6
School’s pupil teacher ratio (deciles)	6.393 (2.576)	6.504 (2.551)	0.23	6.450 (2.563)	1.6
School’s proportion of native English speakers (deciles)	2.567 (1.186)	2.598 (1.187)	0.46	2.583 (1.187)	1.6
School’s proportion of free school meal eligibility (deciles)	5.337 (2.812)	5.198 (2.732)	0.17	5.266 (2.772)	5.8
School’s proportion of children with special educational needs (deciles)	5.318 (2.736)	5.323 (2.802)	0.96	5.320 (2.769)	1.9
School’s average Key Stage 1 score (deciles)	5.631 (2.789)	5.675 (2.850)	0.68	5.653 (2.818)	8.1

Categorical variables	*N* (%)	*N* (%)	*p* value	*N *(%)	% Missing data
Ethnicity, White	1,256 (81.3)	1,307 (81.9)	0.67	2,563 (81.6)	0.0
Mother has university degree	284 (19.3)	275 (18.0)	0.34	559 (18.6)	4.5
Eligible for free school meals	155 (11.1)	191 (13.2)	0.09	346 (12.1)	9.3
Does not have special educational needs	1,041 (67.7)	1,284 (80.6)	<0 .001	2,325 (74.3)	0.3
Lives with both biological parents	1,202 (77.8)	1,240 (77.7)	0.94	2,442 (77.8)	0.0
Shows signs of puberty (at age 11 years)	531 (38.7)	1,445 (99.8)	<0 .001	1,976 (70.1)	10.3
Lives in urban area	1,257 (81.4)	1,316 (82.5)	0.43	2,573 (81.9)	0.0

## Results

Table 1 summarises the sample characteristics and proportion of missing data in the sample. The analytic sample comprised 3,141 children, 1,545 of whom were males (49%) and 1,596 females (51%). The majority of children were white (*N* = 2,563; 82%), lived with both biological parents (*N* = 2,442; 78%), resided in urban areas (*N* = 2,573; 82%) and did not have SEN (*N* = 3,325; 74%). Moreover, 19% (*N* = 559) had mothers with a university degree and 12% (*N* = 346) were eligible for FSM. By age 11 years, 70% were showing signs of puberty. In terms of sex differences, males scored significantly higher in delay aversion and risk-taking, had more externalising problems and were more likely to have SEN (all *p* values < 0.01). Females scored significantly higher in deliberation time, had higher KS1 scores, and were more likely to be showing signs of puberty by age 11 years (all *p* values < 0.01). No other comparisons between sexes yielded statistically significant results.

Table 2 summarises the bivariate associations between the primary school characteristics in the analytic sample. Almost all correlations were statistically significant and reflected an ecology of disadvantage. The strongest (negative) associations were found between school’s average KS1 scores and proportion of children with FSM eligibility (rho = –0.65, *p* < 0.01), and between school’s average KS1 scores and proportion of children with SEN (rho = –0.52, *p* < 0.01). There was a strong positive association of the proportion of children with FSM eligibility with the proportion of children with SEN (rho = 0.57, *p* < 0.01).

**Table 2:** Spearman’s correlation coefficients (rho) between school compositional and structural characteristics

	1	2	3	4	5	6
1.School size (deciles)	1.00					
2.School’s pupil teacher ratio (deciles)	0.260**	1.00				
3.School’s proportion of native English speakers (deciles)	–0.289**	0.021	1.00			
4.School’s proportion of free school meal eligibility (deciles)	0.062**	–0.307**	–0.371**	1.00		
5.School’s proportion of children with special educational needs (deciles)	–0.022	–0.198**	–0.211**	0.571**	1.00	
6.School’s average Key Stage 1 score (deciles)	–0.010**	0.212**	0.264**	–0.617**	–0.522**	1.00

*p < 0.05; **p < 0.01

Tables 3 and 4 summarise the results of the multiple linear regression models performed to assess the role of school characteristics for decision-making in males and females, respectively. In males (Table 3), after adjustments for confounding, school’s proportion of native English speakers was significantly and positively associated with longer deliberation time (*b* = 0.09, *p* < 0.05); school’s higher proportion of children with FSM eligibility was significantly associated with worse quality of decision-making (*b* = –0.01, *p* < 0.05); school’s proportion of children with SEN was positively associated with better quality of decision-making (*b* = 0.01, *p* < 0.05); and school’s average academic performance was negatively associated with delay aversion (*b* = –0.01, *p* < 0.05). Of the covariates considered, increased levels of externalising problems were associated with increased delay aversion (*b* = 0.01, *p* < 0.01); own FSM eligibility with poorer quality of decision-making (*b* = –0.05, *p* < 0.05); and own academic performance with decreased delay aversion (*b* = –0.01, *p* < 0.01), shorter deliberation time (*b* = –0.06, *p* < 0.01), better quality of decision-making (*b* = 0.01, *p* < 0.01) and greater risk adjustment (*b* = 0.06, *p* < 0.01).

**Table 3:** Crude and adjusted unstandardised^a^ regression coefficients (SE) of multiple linear regression models examining the prospective associations of school compositional and structural characteristics with decision-making outcomes before and after adjustments for confounding in males (*N* = 1,545)

	Delay aversion		Deliberation time^b^		Quality of decision-making		Risk adjustment		Risk taking	
	Model A	Model B	Model A	Model B	Model A	Model B	Model A	Model B	Model A	Model B
School compositional characteristics										
School size (deciles)	–.002 (.003)	–.001 (.003)	.014 (.016)	.011 (.016)	.001 (.002)	.002 (.002)	–.008 (.011)	–.006 (.011)	.001 (.002)	.001 (.002)
School’s pupil teacher ratio (deciles)	–.001 (.003)	–.001 (.003)	.003 (.016)	.003 (.015)	–.001 (.002)	–.002 (.002)	.001 (.012)	.001 (.012)	–.001 (.002)	–.001 (.002)
Schools proportion of native English speakers (deciles)	–.005 (.006)	–.006 (.006)	.080 (.032)*	.086 (.034)*	.005 (.005)	.003 (.005)	.012 (.029)	.004 (.027)	–.005 (.005)	–.004 (.005)
School’s proportion of free school meal eligibility (deciles)	–.005 (.004)	–.004 (.003)	.028 (.018)	.020 (.019)	–.008 (.003)**	–.005 (.003)*	–.036 (.017)*	–.026 (.017)	–.002 (.003)	–.002 (.003)
School’s proportion of children with special education needs (deciles)	–.004 (.003)	–.004 (.003)	.016 (.016)	.017 (.016)	.005 (.003)*	.005 (.002)*	.013 (.012)	.015 (.013)	.001 (.002)	.001 (.002)
School’s average Key Stage 1 score (deciles)	–.011 (.003)**	–.007 (.003)*	–.010 (.016)	.016 (.016)	.007 (.002)**	.002 (.003)	.037 (.015)*	.004 (.015)	–.001 (.002)	.000 (.003)
Individual and family characteristics										
Internalising problems	–	.000 (.002)	–	.004 (.014)	–	–.001 (.003)	–	–.004 (.017)	–	–.002 (.002)
Externalising problems	–	.007 (.002)**	–	–0.003 (.010)	–	–.001 (.001)	–	–.002 (.011)	–	.002 (.001)
Shows signs of puberty (at age 11)	–	.003 (.014)	–	.116 (.068)	–	–.010 (.011)	–	–.072 (.062)	–	.011 (.011)
Does not have special educational needs	–	.010 (.016)	–	.064 (.098)	–	.001 (.011)	–	.130 (.084)	–	–.001 (.011)
Mother has university degree	–	.019 (.016)	–	–.007 (.084)	–	.001 (.014)	–	.073 (.099)	–	–.006 (.012)
Average Key Stage 1 score	–	–.008 (.003)**	–	–.059 (.014)**	–	.010 (.002)**	–	.062 (.012)**	–	–.002 (.002)
Maternal psychological distress	–	–.001 (.002)	–	.006 (.011)	–	.000 (.001)	–	.012 (.008)	–	.000 (.001)
Eligible for free school meals	–	–.049 (.029)	–	.223 (.116)	–	–.045 (.019)*	–	–.067 (.130)	–	–.007 (.017)
Lives with both biological parents	–	–.008 (.016)	–	.134 (.084)	–	.002 (.014)	–	.028 (.090)	–	.017 (.012)
Ethnicity, White	–	.009 (.019)	–	–.016 (.104)	–	.022 (.019)	–	.029 (.090)	–	–.008 (.018)
Lives in urban area	–	–.027 (.017)	–	.027 (.101)	–	.007 (.014)	–	–.054 (.081)	–	.006 (.013)

*Note* All models were adjusted for the stratified design of Millennium Cohort Study

^a^The magnitude of the regression coefficients presented should be interpreted according to the range of the corresponding exposure variable

^b^Measured in seconds

*p < 0.05; **p < 0.01

**Table 4:** Crude and adjusted unstandardised ^a^ regression coefficients (SE) of multiple linear regression models examining the prospective associations of school compositional and structural characteristics with decision-making outcomes before and after adjustments for confounding in females (*N* = 1,596)

	Delay aversion		Deliberation time^b^		Quality of decision-making		Risk adjustment		Risk taking	
	Model A	Model B	Model A	Model B	Model A	Model B	Model A	Model B	Model A	Model B
School compositional characteristics										
School size (deciles)	.000 (.004)	.000 (.004)	–.005 (.014)	–.006 (.015)	–.002 (.002)	–.001 (.002)	–.004 (.012)	.005 (.012)	.001 (.002)	.001 (.002)
School’s pupil teacher ratio (deciles)	–.001 (.003)	–.001 (.003)	–.001 (.015)	.002 (.016)	.001 (.002)	.001 (.002)	.020 (.013)	.017 (.013)	–.001 (.002)	–.001 (.002)
Schools proportion of native English speakers (deciles)	.010 (.008)	.009 (.009)	–.051 (.041)	–.058 (.043)	.007 (.004)	.005 (.005)	–.002 (.030)	–.030 (.035)	–.004 (.005)	.002 (.005)
School’s proportion of free school meal eligibility (deciles)	.002 (.004)	.001 (.004)	.029 (.022)	.022 (.024)	.001 (.003)	.002 (.003)	–.035 (.018)*	–.023 (.018)	.002 (.003)	.002 (.003)
School’s proportion of children with special education needs (deciles)	–.001 (.003)	.000 (.003)	–.002 (.017)	–.002 (.016)	.000 (.002)	.000 (.002)	.008 (.014)	.006 (.014)	–.005 (.002)*	–.005 (.002)*
School’s average Key Stage 1 score (deciles)	.000 (.004)	.002 (.004)	.031 (.020)	.059 (.021)**	.005 (.002)*	.002 (.002)	.018 (.013)	–.002 (.015)	–.006 (.002)**	–.005 (.002)*
Individual and family characteristics										
Internalising problems	–	–.004 (.003)	–	.014 (.016)	–	–.001 (.002)	–	.000 (.014)	–	–.003 (.002)
Externalising problems	–	.004 (.003)	–	–.002 (.013)	–	–.003 (.002)	–	–.014 (.012)	–	.001 (.002)
Shows signs of puberty (at age 11)	–	–.028 (.256)	–	–.101 (.162)	–	–.100 (.076)	–	.485 (.390)	–	–.002 (.058)
Does not have special educational needs	–	.003 (.019)	–	.119 (.109)	–	.000 (.014)	–	–.118 (.083)	–	–.010 (.013)
Mother has university degree	–	.012 (.018)	–	–.182 (.104)	–	.037 (.013)**	–	.162 (.072)*	–	–.019 (.012)
Average Key Stage 1 score	–	–.004 (.003)	–	–.069 (.016)**	–	.007 (.003)**	–	.043 (.014)**	–	–.002 (.002)
Maternal psychological distress	–	.002 (.002)	–	.000 (.009)	–	.000 (.001)	–	–.011 (.009)	–	.001 (.002)
Eligible for free school meals	–	.000 (.028)	–	–.104 (.145)	–	.023 (.016)	–	.070 (.095)	–	–.033 (.016)*
Lives with both biological parents	–	–.024 (.018)	–	–.094 (.107)	–	.004 (.013)	–	.041 (.078)	–	–.017 (.012)
Ethnicity, White	–	–.003 (.025)	–	.017 (.119)	–	.016 (.016)	–	.210 (.113)	–	–.053 (.016)**
Lives in urban area	–	–.006 (.021)	–	–.045 (.115)	–	–.005 (.017)	–	–.082 (.082)	–	–.008 (.013)

*Note *All models were adjusted for the stratified design of Millennium Cohort Study

^a^The magnitude of the regression coefficients presented should be interpreted according to the range of the corresponding exposure variable

^b^Measured in seconds

*p <0 .05; **p < 0.01

In females (Table 4), after adjustment for confounding, the school’s proportion of children with SEN was associated negatively with risk-taking (*b* = –0.01, *p* < 0.05), while the school’s average KS1 score was positively associated with deliberation time (*b* = 0.06, *p* = 0.02) and negatively with risk-taking (*b* = –0.01, *p* < 0.05). Of the covariates considered, higher maternal education was associated with improved quality of decision-making (*b* = 0.04, *p* < 0.05) and greater risk adjustment (*b* = 0.16, *p* < 0.05). Own academic performance was associated negatively with deliberation time (*b* = –0.07, *p* < 0.01) and positively with quality of decision-making (*b* = 0.01, *p* < 0.01) and risk adjustment (*b* = 0.04, *p* < 0.01). Finally, own FSM eligibility was associated with less risk-taking (*b* = –0.03, *p* < 0.05). Mean VIF values in Models B in males and females were 1.43 and 1.40, respectively, indicating that none of the predictors were highly collinear (highest VIF value was observed for school’s proportion of FSM eligible among females, VIF = 2.37).^1^

## Discussion

The findings of this study suggest that certain primary school characteristics are associated with individual children’s decision-making. In partial agreement with our hypotheses we found that after adjustment for individual and family-level confounding, those schools with a higher average academic performance showed more delay aversion among individual males, and higher deliberation time and lower risk-taking among individual females. In schools with a higher proportion of native English speakers individual males had higher deliberation time. In schools with a higher proportion of pupils eligible for FSMs individual males showed lower scores on quality of decision-making. Finally, in schools with higher proportions of children with special educational needs individual males showed better quality of decision-making and individual females showed lower risk-taking. Prior to examining these results more closely we note that they are associative and cannot be used to infer causal links between a school’s compositional characteristics and a pupil’s affective decision-making. Future studies aiming to test causal links should attempt to control for pre-existing differences in decision-making and to use mixed-methods approaches to identify additional individual and school-level factors which might be driving the significant associations that we found in this study.

Turning to the results of our study, the fact that the proportion of native English speakers in the school is positively associated with individual pupils’ deliberation time was rather unexpected given the positive impact that interacting with peers of other ethnicities can have on children’s cognitive maturity [18]. Speed of information processing, largely captured by deliberation time on the CGT, is associated with activations of brain areas similar to the ones observed for inhibitory control, a cognitive mechanism that serves to block affective impulses and, therefore, enables deliberative decision-making even in affect-charged situations [50–52]. Howbeit, a school’s proportion of pupils with English as an additional language (EAL) cannot necessarily be considered a good proxy of the ethnic diversity of the student body. Moreover, as discussed in greater detail below, we could not control for native English speaker status at the individual level since the MCS recruited children born in the UK and such analyses would be severely underpowered. This finding should be interpreted with this caveat in mind. Our results also suggest that male pupils in schools with higher average SES show better quality of decision-making, independently of their own SES. Importantly these associations survived adjustments for individual and family-level confounding known to be associated with school selection and decision-making. If these associations were causal, they suggest that interventions aiming to support lower-achieving schools and those with less affluent intakes could help to improve all boys’ decision-making. For females, attending a higher performing school was related to less delay aversion and less risk-taking but also longer deliberation time, independently of their own academic performance. Previous evidence also suggests advantageous effects of such schools on behavioural adjustment and academic performance [4, 53], both of which are closely linked with reduced delay aversion but also quality of decision-making [7, 24]. Regarding the positive association found between attending a higher performing school and one’s deliberation time, we believe that this finding might be an artefact of higher rates of females in schools with a higher average academic performance. Girls have been shown to have longer deliberation times than boys at age 11 years [7] and a disadvantage in processing speed (reaction time) tasks including motor responses, e.g. finger tapping [54]. At the same time, they outperform boys in school [55]. Taken together, this line of evidence suggests that it is likely that better performing schools have a higher proportion of female pupils which might explain the positive association between average school performance and deliberation time among individual female pupils. In the absence of sex ratio data for our schools, we could not test this hypothesis explicitly.

An additional surprising finding of the study was the association between the proportion of pupils in the school with SEN and individual females’ reduced risk-taking and individual males’ better quality of decision-making. An explanation may be that inclusive schools with higher proportions of pupils with SEN tend to be more innovative in their teaching approaches and thus benefit all children. For example, schools with more pupils with SEN which are unable to offer individual support might adopt more creative ways of engaging all children in learning, thus benefitting all pupils’ quality of decision-making either directly or indirectly by enhancing the school climate and cultivating an inclusive school culture [56]. Although we could not determine how inclusive the MCS schools with a higher proportions of children with SEN were, we think that these findings provide support for the benefits of inclusive learning. This is particularly relevant in light of evidence suggesting that pupils with SEN in English schools are disproportionately excluded [57]. At the same time, there is evidence suggesting that all children who attend highly inclusive schools perform better academically and interact more positively with peers compared with pupils educated in low inclusive settings [58]. Our study supports this notion that diversion should be at the heart of inclusion since inclusive learning environments might also benefit aspects of learners’ decision-making.

Our study, however, also has several limitations, as already outlined, and the results should be interpreted with these caveats in mind. First, in the absence of measurements for pre-existing differences in decision-making our results are associative and cannot be used to infer causal links between the school’s compositional characteristics and an individual pupil’s affective decision-making. Nonetheless, by controlling for emotional and behavioural problems and academic performance (at age 7) which are correlated with decision-making [3, 7] it is likely that prior differences were, at least partially, taken into account. Second, not all characteristics measured at the school level were also captured at the individual level in our analyses. For example, EAL at the individual level was excluded due to the small number of non-native English speakers in the MCS given that the cohort had all been living in the UK since infancy. Third, school characteristics were measured in NPD in 2008 and were considered in our study to be invariant until 2011/12 when the MCS children took the CGT. Since we excluded from the analytic sample those children who changed school between the two assessments this assumption is likely to hold to a large extent. Fourth, the analytic sample (*N* = 3,141) consisted of 77% of the total sample of children eligible for our study with available data from the January 2008 census and the outcomes considered (*N *= 4,076). However, we attempted to eliminate biases resulting from attrition and non-response using study-specific weights which can account for the disproportionate attrition of participants in MCS. Fifth, the adequacy of FSM eligibility as a proxy of SES has been questioned in the available literature [59], however, there is also evidence that FSM eligibility comes very close to identifying a disadvantaged group of children [60]. Sixth, the variable used to capture SEN at the school-level excluded children with formal statements but that was not the case for the variable for SEN at the individual-level. Since only 46 children in the analytic sample (1%) had a statement according to the teacher survey this discrepancy is highly unlikely to have influenced the results. Seventh, due to small degree of clustering of pupils within school in our analytic sample we could not take the hierarchical nature of the data into account by modelling children as being nested within schools by means of multilevel models. However, by considering simultaneously characteristics at both the individual and school levels, we avoided committing the ecological fallacy, whereby inference occurs at the group level (school in this case), but is actually attributable to confounding by individual factors [61]. Nonetheless, it is strongly recommended that future studies utilise datasets specifically designed to model school effects by providing an adequate degree for clustering of pupils within schools. Finally, we only found few significant associations between school characteristics and the decision-making outcomes considered, and effect sizes were rather small. Nevertheless, such associations had not been investigated before, and one would not expect them to dominate individual/family-level effects. They may be weak, but they do exist.

## Conclusion

The findings of this study suggest that primary school composition is associated with important aspects of individual children’s decision-making. Pupils in less socioeconomically disadvantaged schools and those in better performing schools were making, on average, better and less impulsive choices, even after controlling for their own level of socioeconomic disadvantage and academic performance. The findings of the study suggest that interventions aiming to support lower-achieving schools and those with less affluent intakes could help to improve decision-making, but mainly in boys.

## Data Availability

This study made use of third-party data and we do not have the rights to share.

## Footnotes

1 In a supplementary analysis we also tested cross-level interactions between school-average KS1 performance, proportion of children in the school eligible for FSM, and proportion of children in the school with SEN, and the equivalent individual characteristics (i.e. child’s KS1 score, child’s FSM eligibility and child’s SEN status) on the decision-making outcomes after adjustments for confounding. None of these interaction terms yielded significant results.
